# High HIV incidence epidemic among men who have sex with men in china: results from a multi-site cross-sectional study

**DOI:** 10.1186/s40249-016-0178-x

**Published:** 2016-09-05

**Authors:** Jun-Jie Xu, Wei-Ming Tang, Hua-Chun Zou, Tanmay Mahapatra, Qing-Hai Hu, Geng-Feng Fu, Zhe Wang, Lin Lu, Ming-Hua Zhuang, Xi Chen, Ji-Hua Fu, Yan-Qiu Yu, Jin-Xin Lu, Yong-Jun Jiang, Wen-Qing Geng, Xiao-Xu Han, Hong Shang

**Affiliations:** 1 Key Laboratory of AIDS Immunology of National Health and Family Planning Commission, Department of Laboratory Medicine, The First Affiliated Hospital, China Medical University, Shenyang, 110001 People’s Republic of China; 2 Collaborative Innovation Center for Diagnosis and Treatment of Infectious Diseases, Hangzhou, People’s Republic of China; 3 University of North Carolina at Chapel Hill, Project-China, No. 2 Lujing Road, Guangzhou, 510085 People’s Republic of China; 4 Guangdong Provincial Center for Skin Diseases and STIs Control, Guangzhou, 510085 People’s Republic of China; 5 Kirby Institute, the University of New South Wales, Sydney, NSW 2052 Australia; 6 Department of Epidemiology, Fielding School of Public Health, University of California, Los Angeles, 90095 USA; 7 Jiangsu Provincial Centers for Disease Control and Prevention, Nanjing, China; 8 He’nan Provincial Centers for Disease Control and Prevention, Zhengzhou, China; 9 Yunnan Provincial Centers for Disease Control and Prevention, Kunming, China; 10 Shanghai Municipal Centers for Disease Control and Prevention, Shanghai, China; 11 Hu’nan Provincial Centers for Disease Control and Prevention, Changsha, China; 12 Shandong Provincial Centers for Disease Control and Prevention, Jinan, China

**Keywords:** Men who have sex with men (MSM), HIV, Incidence, BED HIV-1 capture enzyme immunoassay (BED-CEIA)

## Abstract

**Background:**

Recent upsurge of new HIV infections among men who have sex with men (MSM) is a major concern in China. Paucity of national-level information regarding the burden and predictors of this progressive epidemic of new infections called for a multi-centric, timely and comprehensive investigation.

**Methods:**

Mixed methods were used to recruit MSM from seven cities in China between 2012 and 2013. Recent and established HIV infections were estimated by Western Blot and BED HIV-1 capture enzyme immunoassay. Syphilis and herpes simplex virus-2 (HSV-2) were also tested.

**Results:**

A total of 4496 eligible MSM were recruited. The majority was aged ≤35 years (77.5 %), migrants (60.3 %), never married (69.8 %), and played receptive role in anal sex (70.5 %). The HIV prevalence was 9.9 %, and 41.9 % were recently infected, with sensitivity/specificity adjusted HIV incidence of 8.9 (95 % *CI*: 7.6-10.2)/100 Person-Years. The prevalence of history HSV-2 and syphilis were 12.5 % and 8.5 %, respectively. Recent HIV infection was associated with having multiple male partners (a*OR* = 1.4, 95 % *CI* 1.1-1.9), recreational drug use (a*OR* = 2.2, 95 % *CI* 1.6-3.0), anal bleeding (a*OR* = 2.1, 95 % *CI* 1.4-3.0), syphilis infection (a*OR* = 2.8, 95 % *CI* 1.9-4.3) and history HSV-2 infection (a*OR* = 2.3, 95 % *CI* 1.5-3.3).

**Conclusion:**

High rate of recent HIV infection is potentially resulting in progressive deterioration of the overall HIV epidemic among MSM in China. Targeted interventions to address high-risk MSM including those having multiple partners, history of recreational drug use and syphilis or HSV-2 infection seemed to be the need of the hour.

**Electronic supplementary material:**

The online version of this article (doi:10.1186/s40249-016-0178-x) contains supplementary material, which is available to authorized users.

## Multilingual abstracts

Please see Additional file 1 for translations of the abstract into the six official working languages of the United Nations.

## Background

The current HIV situation among men who have sex with men (MSM) in China is a major public health concern as in the upsurge of the epidemic through sexual route in the past decade (in 2011, 76.3 % were infected through sexual route as opposed to 33.1 % in 2006) in this country [1]. Simultaneously, sexual relationship among males played an important role in driving this change (from 7.3 % in 2005 to 16.1 % in 2011) [1]. In the early days of HIV epidemic in China, the disease was largely limited to injecting drug users and former plasma donors [2, 3]. In the past decade the epidemic has gradually concentrated in MSM. In 2009 approximately 8.6 % of existing HIV infections were transmitted through male-to-male sexual route and this figure increased to 21.4 % in 2013 [1].

It seems to be getting more complicated with the progressive rise in new infections in this grossly understudied population possibly due to several unidentified and yet unaddressed risky sexual behaviors [4]. The proportion of MSM among recently identified HIV cases has been increasing at an alarming rate (29.4 % in 2009 vs. 12.2 % in 2007) [1]. These gradually progressive new HIV infections among MSM can cumulatively result in an explosive upsurge of HIV epidemic in this country. More importantly, recently infected HIV cases usually have very high viral load and thus very highly infectious although they often remain undetected especially due to lack of pronounced symptoms [5]. MSM engaged in anal intercourse with recently infected HIV cases in their community have a very high risk of HIV transmission. In addition, MSM population is known to exhibit several high risk behaviors like having multiple partners, non-use of condoms, being engaged in unprotected sex, use of drugs, etc. Each of which increases the chances of HIV transmission from and within this vulnerable population.

Despite all these potential vulnerabilities for HIV acquisition and transmission among MSM, given the fact that the risk of missing recent HIV infections from the coverage of HIV prevention and treatment programs, studies to determine potential predictors of recent HIV infection among MSM were handful. The limited available evidences dealt only with some dispersed communities in specific locations of this huge country [6] while there was not much effort to measure the occurrence of recent HIV infection in a comprehensive, multi-centric approach using prospective design. To fill this knowledge gap, we conducted a detailed evaluation involving a relatively large MSM population from different parts of the country.

## Methods

### Study design and objectives

In order to understand the situation of recent and established HIV infection among Chinese MSM, a comprehensive multisite cross-sectional study was conducted in seven cities of China (Shanghai, Nanjing, Changsha, Zhengzhou, Ji’nan, Shenyang and Kunming) between June 2012 and June 2013, to measure the incidence of recent HIV infections.

### Study participants

In our study, cruising areas and service points (sexually transmitted disease (STD) clinics etc.) for MSM were used as the sampling sites. Site-specific sampling periods were determined based on attendance and hours of operation. Men who attended the sites during the study period, had sex with men (oral and/or anal) within last one year, aged 18 years or older, never being tested as HIV positive before, and provided written informed consent were recruited at the scheduled sites. Participants with first CD4^+^ T cell counts <350/mm^3^, or had any AIDS-defining illness were also excluded.

Three methods were used for recruitment. (a): Through internet: The study was introduced and promoted in the discussion forums of local gay websites or online chat rooms by posting the IRB-approved introduction material and eligible participants were enrolled directly. (b): Venue-based sampling: With the help of local MSM community based organizations, eligible MSM were also recruited from venues such as gay bars, parks, and bathhouses visited by local MSM. (c): Peer referrals: MSM Participants were encouraged to introduce and recruit their peer male partners or MSM friends to the survey.

### Structured interview

Eligible participants were interviewed face-to-face using a structured questionnaire to collect information on socio-demographics, AIDS related knowledge, recent sexual behavior, history of drug use and health seeking behaviors. Demographic information included age (18-25/26-35/36-45/≥46), marital status (never married to a woman/ever married to a women), educational level (elementary school or lower/junior high school/senior high school/college or higher), residency status (official/non-official resident of the sampling cities), self-identified sexual orientation (homosexual/bisexual) and cruising venues (Internet/others). HIV prevention related knowledge (adequate/inadequate) of the participants was assessed by asking eight relevant questions. Having adequate knowledge was defined by the ability to provide appropriate responses to all the questions.

Participants were asked about role in anal sex (insertive only/receptive only/both) and number of male partners in the past six months (≤2/>2). Additional information was also collected on history of unprotected anal (UAI) and vaginal sex (UVI) in past six month (yes/no), use of any type of recreational drugs ever (such as rush (poppers or alkyl nitrites), ecstasy, ice, amphetamine, tramadol, and ketamine, etc.) (yes/no), any experience of condom breakage and slippage during sex in the last six months (yes/no) and circumcision status.

### Serological measures

Venous blood samples were collected from participants for serological antibody tests for diagnosing HIV, syphilis, herpes simplex virus-2 (HSV-2). Rapid plasma reagin [RPR] test (Shanghai Kehua, China) was used to screen syphilis and positive tests were confirmed by Treponema pallidum particle assay (TPPA) (Hainan Huamei, China). HSV-2 infection was determined using ELISA (HerpeSelect-2, Focus Technologies, USA). Screening for HIV-1 antibody was conducted by enzyme-linked immunosorbent assay [ELISA] (Vironostika HIV-1 Microelisa System; bioMerieux, Durham, NC) and positive cases were confirmed by Western Blotting (HIV Blot 2.2 WBTM, Genelabs Diagnostics, Singapore).

**Fig. 1:**
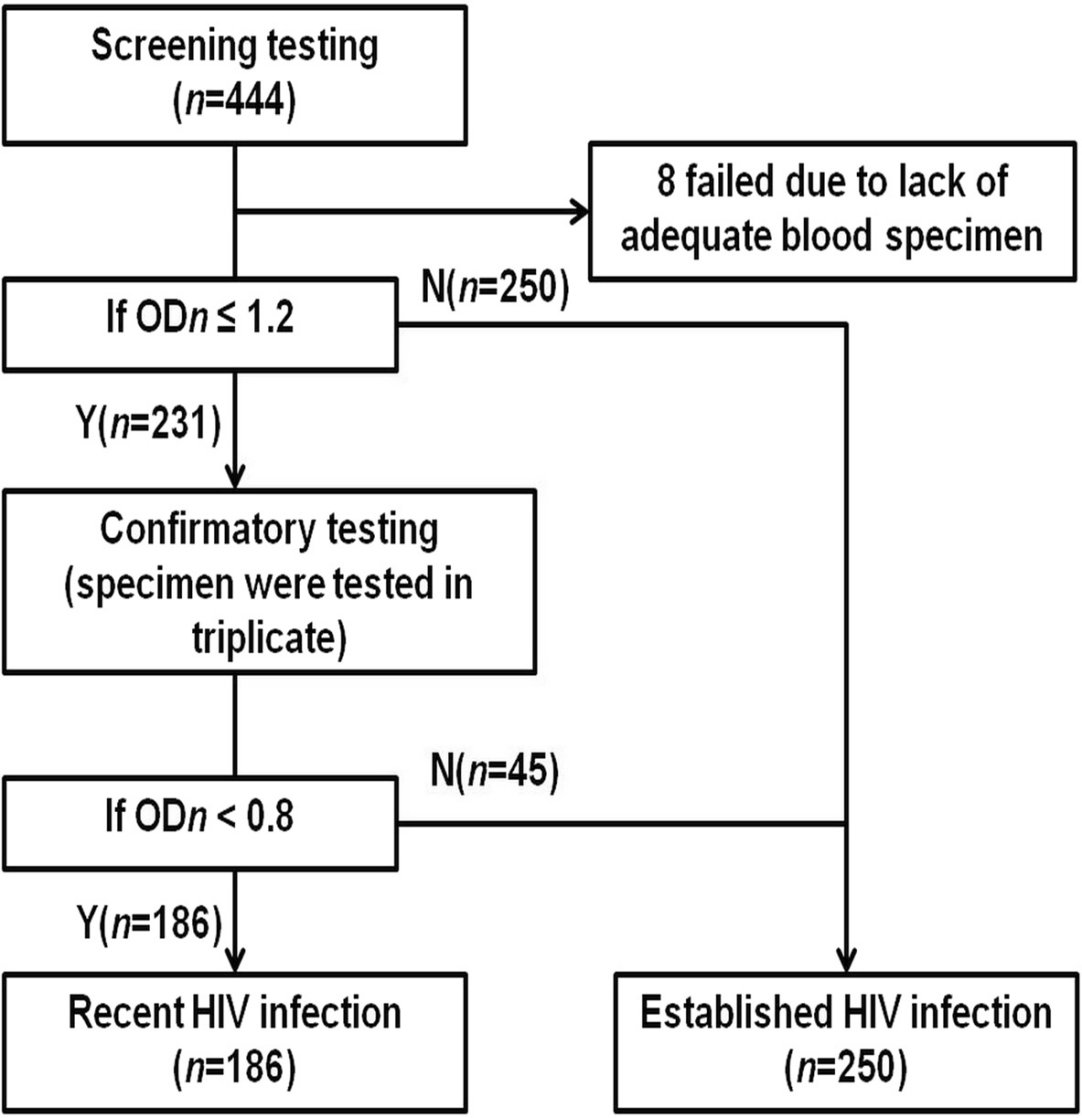
Flowchart of BED-CEIA testing of HIV recent infection

All Western blot-positive samples were also tested by BED HIV-1 capture enzyme immunoassay (BED-CEIA, Calypte Biomedical Corporation, Rockville, MD, USA). BED-CEIA is a popular method that can be used to determine the incidence of HIV based on cross-sectional survey among specific populations with high risk of HIV infection [7, 8]. HIV-1 BED-CEIA was tested at the Key Laboratory of AIDS Immunology of National Health and Family Planning Commission in Shenyang, China by trained technicians (Fig. 1). Specimens with initial ODn >1.2 were classified as established HIV infection. Specimens with initial ODn <1.2 were tested in triplicate to confirm their ODn values. If the median ODn value from all three tests was <0.8, the HIV case was considered as recently infected (<168 days, which we defined as recent infection in based on China HIV reference lab estimated HIV recent infection window period [9], otherwise, the HIV infection was classified as established infection.

Samples were optimally stored in low temperature laboratory freezer (minus eighty degrees Celsius) before carrying out BED-CEIA test, and drikold were used to keep the low temperature of the specimens prior to distribution to the Shenyang central laboratory for BED-CEIA testing. Antibody tests for HIV, syphilis, HSV-2 were conducted in respective provincial HIV laboratories of CDC to which the seven study sites were affiliated.

### Data analysis

Data were double-entered and logic checked using EpiData 3.0. Descriptive statistics were used to describe demographic characteristics, sexual behaviors and prevalence of infections. Based on the BED-CEIA test results, crude incidence of HIV was estimated using the formula (recommended by CDC, Atlanta, USA): Incidence $I=\frac{\left(365/w\right)R}{N+\left(365/w\right)\left(R/2\right)}\times 100$, where w is the Chinese specific window period (168 days) [9], R is the total tested recent HIV infections in the BED-CEIA, and N is the total tested number of HIV-seronegative participants. We also adjusted for false positive rate (FPR) of recent infection testing algorithm (RITA) by using the Sensitivity/Specificity Adjustment formula: $\mathrm{I} = \frac{\left(\mathrm{F}\right)\times \left(365/w\right)R}{N+(F)\left(365/w\right)\left(R/2\right)}$ , where *F* = Correction Factor, and $F=\frac{\left(\mathrm{R}/\mathrm{P}\right) + \gamma -1}{\left(R/P\right)\left(\alpha -\beta +2\gamma -1\right)}$ in which w = 168 days, α = 0.8098, β = 0.7571, γ = 0.9315, FPR = R/P = 0.0685, where P is the total number of cases of longstanding infection in the survey used for estimation of the FRR, and R is the number of these specimens classified as recent by the BED-CEIA. All these values were calculated based on the testing results of specimens with known date of HIV seroconversion by National HIV Reference Laboratory, National Center for AIDS/STD Control and Prevention, Chinese CDC.

Bivariate and multivariate logistic regression models were used to determine factors associated with recent HIV infection. Potential confounding factors such as age (continuous), study site (Shanghai, Nanjing, Changsha, Zhengzhou, Ji’nan, Shenyang and Kunming), residence (local/migrants), education (illiterate/attended primary school, junior high school, senior high school or equal/college or above) and marital status (never married/ever married), were adjusted for in the multivariate models. SAS version 9.1 (SAS Institute, Cary NC) was used for analysis.

## Results

**Fig. 2:**
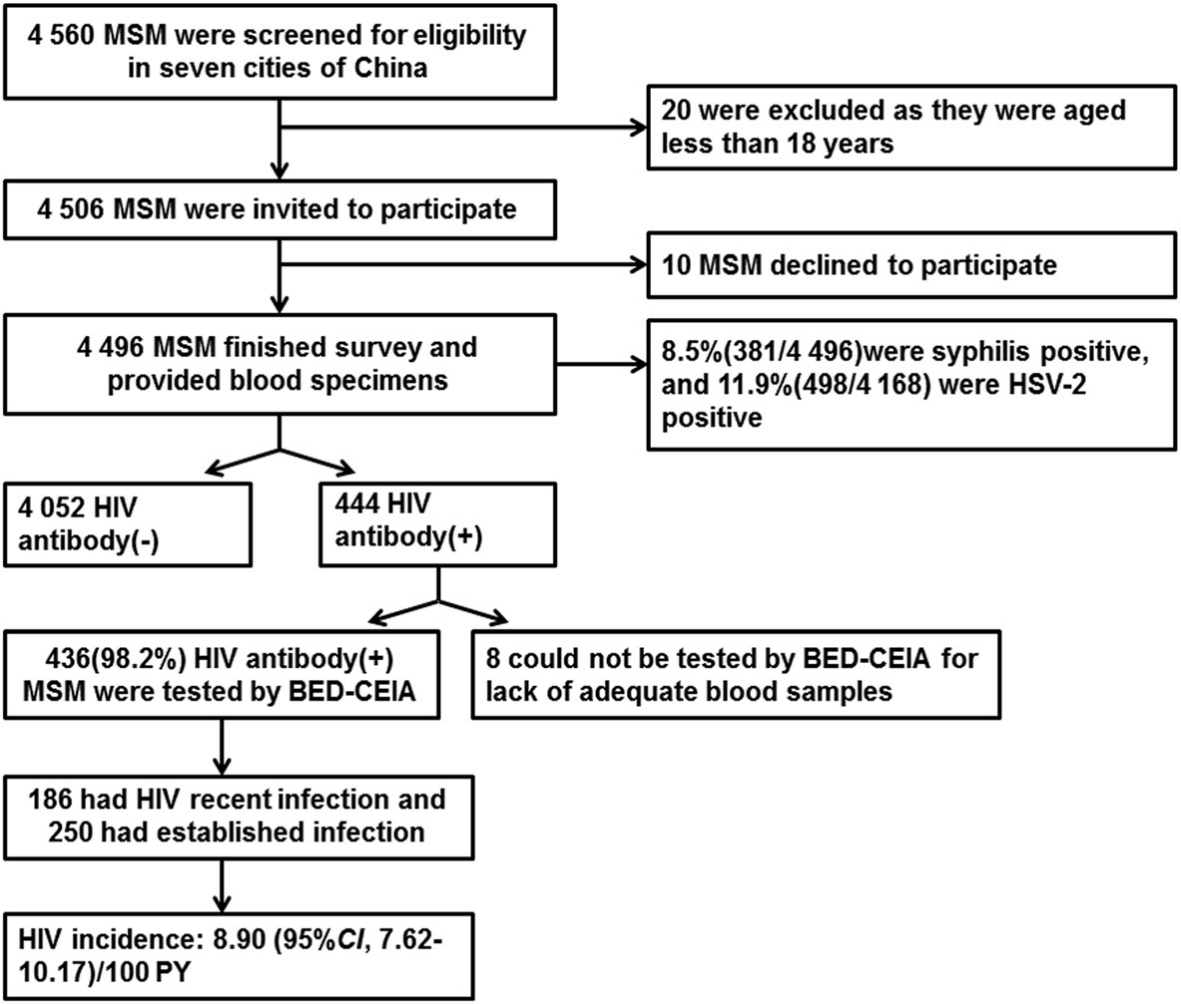
Flowchart of HIV incidence study among MSM in seven cities of China

Between 2012 and 2013, 4 506 eligible MSM were approached and 4 496 (99.8 %) were recruited (Fig. 2).

### Demographics and behaviors

Among the 4 496 total participants, 777(17.3 %), 592(13.2 %), 689(15.3 %), 467(10.4 %), 675(15.0 %), 664(14.8 %) and 632(14.1 %) were recruited from Shanghai, Nanjing, Changsha, Zhengzhou, Ji’nan, Shenyang and Kunming, respectively. In our study, 77.5 % were aged 35 or less, 60.3 % were migrants, 52.7 % attended college or above, 59.2 % self-identified their sexual orientation as homosexual. In addition, 69.8 % of the participants were not married, and 37.5 % had a monthly income of 600 USD or higher (Table 1).

**Table 1:** Distribution of the socio-demographics and HIV-related risk behavior among MSM in China, 2012–2013 (*N* = 4488^a^)

Variables	HIV antibody negative MSM		MSM with established HIV infection		MSM with recent HIV infection		All MSM	
	Nn^b^	Percentage (95 % *CI*)	Nc^b^	Percentage (95 % *CI*)	Ne^b^	Percentage (95 % *CI*)	Nt^b^	Percentage (95 % *CI*)
City								
Kunming	553	13.7 (12.6-14.7)	41	16.4 (11.8-21.0)	37	19.9 (14.1-25.7)	631	14.1 (13.0-15.1)
Shenyang	612	15.1 (14.0-16.2)	30	12.0 (7.9-16.1)	22	11.8 (7.1-16.5)	664	14.8 (13.8-15.8)
Ji’nan	592	14.6 (13.5-15.7)	39	15.6 (11.1-20.1)	43	23.1 (17.0-29.2)	674	15.0 (14.0-16.1)
Changsha	601	14.8 (13.7-15.9)	46	18.4 (13.6-23.2)	38	20.4 (14.6-26.3)	685	15.3 (14.2-16.3)
Zhengzhou	402	9.9 (9.0-10.8)	46	18.4 (13.6-23.2)	17	9.1 (5.0-13.3)	465	10.4 (9.5-11.3)
Nanjing	546	13.5 (12.4-14.5)	30	12.0 (7.9-16.1)	16	8.6 (4.5-12.7)	592	13.2 (12.2-14.2)
Shanghai	746	18.4 (17.2-19.6)	18	7.2 (4.0-10.4)	13	7.0 (3.3-10.7)	777	17.3 (16.2-18.4)
Age								
18-25	1 458	36.0 (34.5-37.5)	71	28.4 (22.8-34.0)	88	47.3 (40.1-54.6)	1617	36.0 (34.6-37.4)
26-35	1 672	41.3 (39.7-42.8)	124	49.6 (43.4-55.8)	66	35.5 (28.5-42.4)	1862	41.5 (40.0-42.9)
36-45	580	14.3 (13.2-15.4)	27	10.8 (6.9-14.7)	22	11.8 (7.1-16.5)	629	14.0 (13.0-15.0)
> =46	342	8.4 (7.6-9.3)	28	11.2 (7.3-15.1)	10	5.4 (2.1-8.6)	380	8.5 (7.7-9.3)
Residence								
Local cities	1 613	39.8 (38.3-41.3)	107	42.8 (36.6-49.0)	63	33.9 (27.0-40.7)	1783	39.7 (38.3-41.2)
Non-local cities	2 439	60.2 (58.7-61.7)	143	57.2 (51.0-63.4)	123	66.1 (59.3-73.0)	2705	60.3 (58.8-61.7)
Education								
Primary school or below	138	3.4 (2.8-4.0)	9	3.6 (1.3-5.9)	9	4.8 (1.7-8.0)	156	3.5 (2.9-4.0)
Junior school	675	16.7 (15.5-17.8)	50	20.0 (15.0-25.0)	34	18.3 (12.7-23.9)	759	16.9 (15.8-18.0)
High school	1 080	26.7 (25.3-28.0)	72	28.8 (23.1-34.5)	44	23.7 (17.5-29.8)	1196	26.7 (25.4-27.9)
College or above	2 159	53.3 (51.7-54.8)	119	47.6 (41.4-53.8)	99	53.2 (46.0-60.5)	2377	53.0 (51.5-54.4)
Sexual orientation								
Other orientation	1 690	41.7 (40.2-43.2)	82	32.8 (26.9-38.7)	60	32.3 (25.5-39.0)	1832	40.8 (39.4-42.3)
Homosexual orientation	2 362	58.3 (56.8-59.8)	168	67.2 (61.3-73.1)	126	67.7 (61.0-74.5)	2656	59.2 (57.7-60.6)
Marital status								
Ever married	1 210	29.9 (28.5-31.3)	94	37.6 (31.6-43.6)	52	28.0 (21.4-34.5)	1356	30.2 (28.9-31.6)
Never married	2 842	70.1 (68.7-71.5)	156	62.4 (56.4-68.4)	134	72.0 (65.5-78.6)	3132	69.8 (68.4-71.1)
Occupations								
Non-students	3 524	87.0 (85.9-88.0)	239	95.6 (93.0-98.2)	160	86.0 (81.0-91.1)	3923	87.4 (86.4-88.4)
Students	528	13.0 (12.0-14.1)	11	4.4 (1.8-7.0)	26	14.0 (8.9-19.0)	565	12.6 (11.6-13.6)
Monthly income (USD)								
No income	716	17.7 (16.5-18.8)	21	8.4 (4.9-11.9)	31	16.7 (11.3-22.1)	768	17.1(16.0-18.2)
1-600	1 822	45.0 (43.4-46.5)	129	51.6 (45..4-57.8)	86	46.2 (39.0-53.5)	2037	45.4 (43.9-46.8)
> =600	1 514	37.4 (35.9-38.9)	100	40.0 (33.9-46.1)	69	37.1 (30.1-44.1)	1683	37.5 (36.1-38.9)
Main venue of seeking male sexual partners								
Non-Internet	1 303	32.2 (30.7-33.6)	80	32.0 (26.2-37.8)	56	30.1 (23.5-36.8)	1439	32.1 (30.7-33.4)
Internet	2 749	67.8 (66..4-69.3)	170	68.0 (62.2-73.8)	130	69.9 (63.2-76.5)	3049	67.9 (66.6-69.3)
Knowledge about prevention of HIV								
Inadequate	2 258	55.7 (54.2-57.3)	140	56.0 (49.8-62.2)	77	41.4 (34.3-48.5)	2475	55.2 (53.7-56.6)
Adequate	1 794	44.3 (42.7-45.8)	110	44.0 (37.8-50.2)	109	58.6 (51.5-65.7)	2013	44.9 (43.4-46.3)
Age of initial sex(years)								
15 or less	264	6.5 (5.8-7.3)	14	5.6 (2.7-8.5)	7	3.8 (1.0-6.5)	285	6.4 (5.6-7.1)
16-25	3 448	85.1 (84.0-86.2)	208	83.2 (78.5-87.9)	169	90.9 (86.7-95.0)	3825	85.2 (84.2-86.3)
> =26	340	8.4 (5.9-11.8)	28	11.2 (4.0-27.8)	10	5.3 (0.6-34.8)	378	8.5 (7.4-9.4)
Gender of initial sexual partner								
Female	1 404	34.6 (33.2-36.1)	98	39.2 (33.1-45.3)	63	33.9 (27.0-40.7)	1565	34.9 (33.5-36.3)
Male	2 648	65.4 (63.9-66.8)	152	60.8 (54.7-66.9)	123	66.1 (59.3-73.0)	2923	65.1 (63.7-66.5)
Anal sexual experience in last 6-months								
No	248	6.1 (5.4-6.9)	12	4.8 (2.1-7.5)	5	2.7 (0.3-5.0)	265	5.9(5.2-6.6)
Yes	3 804	93.9 (93.1-94.6)	238	95.2 (92.5-97.9)	181	97.3 (95.0-99.7)	4223	94.1 (93.4-94.8)
Predominant sex position in last 6 months								
Receptive or both	2 828	69.8 (68.4-71.2)	190	76.0 (70.7-81.3)	147	79.0 (73.1-84.9)	3165	70.5 (69.2-71.9)
Insertive	1 224	30.2 (28.8-31.6)	60	24.0 (18.7-29.3)	39	21.0 (15.1-26.9)	1323	29.5 (28.1-30.8)
Used condom at last anal sex with male partners								
No	1 111	27.4 (26.0-28.8)	82	32.8 (26.9-38.7)	57	30.6 (24.0-37.3)	1250	27.9 (26.5-29.2)
Yes	2 941	72.6 (71.2-74.0)	168	67.2 (61.3-73.1)	129	69.4 (62.7-76.0)	3238	72.1 (70.8-73.5)
No. of male sexual partners in last six months								
< =2	2 514	62.0 (60.5-63.5)	139	55.6 (49.4-61.8)	100	53.8 (46.5-61.0)	2753	61.3 (59.9-62.8)
> 2	1 538	38.0 (36.5-39.5)	111	44.4 (38.2-50.6)	86	46.2 (39.0-53.5)	1735	38.7 (37.2-40.1)
Having female sexual partners in last 6 months								
No	3 305	81.6 (80.4-82.8)	195	78.0 (72.8-83.2)	158	84.9 (79.8-90.1)	3658	81.5(80.4-82.6)
Yes	747	18.4 (17.2-19.6)	55	22.0 (16.8-27.2)	28	15.1 (9.9-20.2)	830	18.5 (17.4-19.6)
Recreational drug use in last 6 months								
No	2 939	72.5 (71.2-73.9)	162	64.8 (58.8-70.8)	116	62.4 (55.3-69.4)	3217	71.7 (70.4-73.0)
Yes	1 113	27.5 (26.1-28.8)	88	35.2 (29.2-41.2)	70	37.6 (30.6-44.7)	1271	28.3 (27.0-29.6)
STDs-related symptoms in last year								
No	3 759	92.8 (92.0-93.6)	218	87.2 (83.0-91.4)	165	88.7 (84.1-93.3)	4142	92.3 (91.5-93.1)
Yes	293	7.2 (6.4-8.0)	32	12.8 (8.6-17.0)	21	11.3(6.7-15.9)	346	7.7(6.9-8.5)
Anal bleeding in last 6 months								
No	3 454	85.2 (84.1-86.3)	203	81.2 (76.3-86.1)	137	73.7 (67.3-80.0)	3794	84.5 (83.5-85.6)
Yes	598	14.8 (13.7-15.9)	47	18.8 (13.9-23.7)	49	26.3(20.0-32.7)	694	15.5(14.4-16.5)
Condom breakage during anal intercourse in last 6-months								
No	3 700	91.3 (90.4-92.2)	235	94.0 (91.0-97.0)	164	88.2 (83.5-92.9)	4099	91.3 (90.5-92.2)
Yes	352	8.7 (7.8-9.6)	15	6.0 (3.0-9.0)	22	11.8 (7.1-16.5)	389	8.7 (7.8-9.5)
Circumcision experience								
No	3 722	91.9 (91.0-92.7)	226	90.4 (86.7-94.1)	160	86.0 (81.0-91.1)	4108	91.5 (90.7-92.3)
Yes	330	8.1 (7.3-9.0)	24	9.6 (5.9-13.3)	26	14.0 (8.9-19.0)	380	8.5 (7.7-9.3)

^a^Eight of the 444 HIV antibody positive MSM participants were not tested by BED-CEIA due to lack of adequate blood samples

^b^Nn = Number of HIV negative participants, Nc = Number of cases with established HIV infection, Ne = Number of cases with recent/established HIV infection, Nt = Total number of participants tested

Nearly 70 % mainly used internet to find male partners and 91.5 % had their sexual debut before 25 years old. In the last six month, 38.7 % had multiple partners, 94.1 % had engaged in anal sex, 70.5 % preferred to be in receptive role during anal intercourse. Nearly 30 % did not use condom in last anal sex and 90.6 % did not use condom in the last oral sex. In the past six months, 15.5 % experienced anal bleeding, 8.7 % experienced condom breakage, 28.3 % used recreational drugs and 8.5 % were circumcised.

### Sero-status of studied MSM participants

#### HIV prevalence and incidence

In our study, a total of 444 participants were tested positive for HIV, with an HIV prevalence of 9.9 % (95 % *CI*: 4.0 %-13.9 %). Among these HIV-infected men, 250 (56.3 %, 95 *CI*:51.7 %-60.9 %) were identified as established HIV positive cases, while other 186 cases were identified as recently infected (41.9 %, 95 *CI*: 37.3 %-46.5 %). Eight (1.8 %, 95 *CI*: 0.6 %-3.0 %) HIV antibody positive participants failed to be tested by BED-CEIA due to lack of adequate blood samples. BED-CEIA based crude overall HIV incidence was 9.7/100 Person-Years (PYs) (95 % *CI*: 8.3-11.1), and the sensitivity/specificity adjusted HIV incidence was 8.9 (95 % *CI*: 7.6-10.2)/100 PY. The BED-CEIA based adjusted HIV incidence rates for Shanghai, Nanjing, Changsha, Zhengzhou, Ji’nan, Shenyang and Kunming were 3.4, 5.4, 12.5, 7.4, 14.1, 6.9 and 12.9/100PYs, respectively (Table 2).

**Table 2:** HIV, syphilis and HSV-2 prevalence and BED-CEIA based HIV incidence among men who have sex with men in China, 2012–2013 (*N* = 4496)

Study sites	Sero-positive number and prevalence (%)			BED-CEIA HIV incidence	
	HIV(*n*, %)	Syphilis (*n*, %)	HSV-2 (*n*, %)^a^	Crude incidence (95 % *CI*)	Adjusted incidence^b^ (95 % *CI*)
Kunming	79 (12.50)	35 (5.54)	90 (14.2)	13.7 (9.3-18.1)	12.9 (8.7-17.0)
Shenyang	52 (7.83)	65 (9.79)	98 (16.7)	7.5 (4.4-10.7)	6.9 (4.0-9.8)
Ji’nan	83 (12.30)	78 (11.56)	61 (9.1)	14.8 (10.4-19.2)	14.1 (9.9-18.3)
Changsha	88 (12.77)	57 (8.27)	98 (14.2)	13.4 (9.2-17.7)	12.5 (8.5-16.5)
Zhengzhou	65 (13.92)	30 (6.42)	53 (11.3)	9.1 (4.7-13.4)	7.4 (3.9-11.0)
Nanjing	46 (7.77)	63 (10.64)	64 (10.8)	6.2 (3.1-9.2)	5.4( 2.8-8.1)
Shanghai	31 (3.99)	53 (6.82)	88 (11.3)	3.7 (1.7-5.7)	3.4 (1.6-5.2)
Total	444 (9.88)	381 (8.47)	552 (12.5)	9.7 (8.3-11.1)	8.9 (7.6-10.2)

^a^81(1.8 %) of the total 4496 participants failed test for HSV-2 antibody for lack of sufficient blood specimens

^b^Sensitivity/specificity adjusted HIV incidence

#### HSV-2 and syphilis prevalence

A total of 4415 (98.2 %) participants were tested for HSV-2, with an HSV-2 prevalence of 12.5 %. In addition, 381 participants were tested for syphilis positive, determining a syphilis prevalence of 8.5 % (Table 3).

**Table 3:** Bivariate and multivariate analyses to determine the associations between potential predictors and recent HIV infection among participating MSM in seven Chinese cities (*N* = 4238^a^)

Variable				Crude Model		Adjusted Model^b^	
	*N*	Recent HIV infection		c*OR *(95 % *CI*)	*P* Value	a*OR *(95 % CI)	*P* Value
		Frequency	%				
Sexual orientation							
Other orientation	1 750	60	3.4	*Ref*		*Ref*	
Homosexual orientation	2 488	126	5.1	1.5 ( 1.1 ~ 2.1 )	0.01	1.6 ( 1.1 ~ 2.2 )	0.01
Occupations							
Non-students	3 684	160	4.3	*Ref*		*Ref*	
Students	554	26	4.7	1.1 ( 0.7 ~ 1.7 )	0.71	0.8 ( 0.5 ~ 1.3 )	0.30
Monthly income (USD)							
No income	747	31	4.2	*Ref*		*Ref*	
1-600	1 908	86	4.5	1.1 ( 0.7 ~ 1.7 )	0.69	1.5 ( 1.0 ~ 2.4 )	0.06
> = 600	1 583	69	4.4	1.1 ( 0.7 ~ 1.8)	0.82	1.6 ( 1.0 ~ 2.6 )	0.06
Ethnics							
Non-Han	276	12	4.4	*Ref*		*Ref*	
Han	3 962	174	4.4	1.0 ( 0.6 ~ 1.8 )	0.97	1.1 ( 0.6 ~ 2.1 )	0.74
Main venue of seeking male sexual partners							
Non-Internet	1 359	56	4.1	*Ref*		*Ref*	
Internet	2 879	130	4.5	1.1 ( 0.8 ~ 1.5 )	0.56	1.0 ( 0.7 ~ 1.4 )	0.98
Knowledge about prevention of HIV							
Inadequate	2 335	77	3.3	*Ref*		*Ref*	
Adequate	1 903	109	5.7	1.8 ( 1.3 ~ 2.4 )	<0.01	1.4 ( 1.0 ~ 2.0 )	0.04
Age of initial sex (years)							
0-15	271	7	2.6	*Ref*		*Ref*	
16-25	3 617	169	4.7	1.9 ( 0.9 ~ 4.0 )	0.12	1.5 ( 0.7 ~ 3.3 )	0.30
> = 26	350	10	2.9	1.1 ( 0.4 ~ 3.0 )	0.84	1.0 ( 0.4 ~ 2.8 )	0.98
Gender of initial sexual partner							
Female	1 467	63	4.3	*Ref*		*Ref*	
Male	2 771	123	4.4	1.0 ( 0.8 ~ 1.4 )	0.83	1.0 ( 0.7 ~ 1.5 )	0.84
Anal sexual experience in last 6 months							
No	253	5	2.0	*Ref*		*Ref*	
Yes	3 985	181	4.5	2.4 ( 1.0 ~ 5.8 )	0.06	2.4 ( 1.0 ~ 5.9)	0.06
Predominant sex position in last 6 months							
Receptive only or both	2 975	147	4.9	*Ref*		*Ref*	
Insertive only	1 263	39	3.1	0.6 ( 0.4 ~ 0.9 )	0.01	0.5 ( 0.4 ~ 0.8 )	<0.01
Used condom at last anal sex with male partners							
No	1 168	57	4.9	*Ref*		*Ref*	
Yes	3 070	129	4.2	0.9 ( 0.6 ~ 1.2 )	0.34	0.9( 0.7 ~ 1.3 )	0.52
No. of male sexual partners in last 6 months							
< =2	2 614	100	3.8	*Ref*		*Ref*	
> 2	1 624	86	5.3	1.4 ( 1.1 ~ 1.9 )	0.02	1.4 ( 1.1 ~ 1.9 )	0.02
Used condom at last oral sex with male partners							
No	3 841	169	4.4	*Ref*		*Ref*	
Yes	397	17	4.3	1.0 ( 0.6 ~ 1.6 )	0.91	0.9 ( 0.5 ~ 1.5 )	0.66
Having foreign male sexual partners in last 6 months							
No	4 188	181	4.3	*Ref*		*Ref*	
Yes	50	5	10.0	25 ( 1.0 ~ 63 )	0.06	1.8 ( 0.7 ~ 4.7 )	0.220
Having female sexual partners in last 6 months							
No	3 463	158	4.6	*Ref*		*Ref*	
Yes	775	28	3.6	0.8 ( 0.5 ~ 1.2 )	0.24	0.8 ( 0.5 ~ 1.3 )	0.33
Recreational drug use in last 6 months							
No	3 055	116	3.8	*Ref*		*Ref*	
Yes	1 183	70	5.9	1.6 ( 1.2 ~ 2.2 )	0.01	2.2 ( 1.6 ~ 3.0 )	<0.01
STDs-related symptoms in last year							
No	3 924	165	4.2	*Ref*		*Ref*	
Yes	314	21	6.7	1.6 ( 1.0 ~ 2.6 )	0.04	1.7 ( 1.1 ~ 2.8 )	0.03
Anal bleeding in the last 6 months							
No	3 591	137	3.8	*Ref*		*Ref*	
Yes	647	49	7.6	2.1 ( 1.5 ~ 2.9 )	<0.01	2.1 ( 1.4 ~ 3.0 )	<0.01
Condom breakage during anal intercourse in last 6 months							
No	3 864	164	4.2	*Ref*		*Ref*	
Yes	374	22	5.9	1.4 ( 0.9 ~ 2.2 )	0.14	1.5 ( 0.9 ~ 2.4 )	0.11
Circumcision experience							
No	3 882	160	4.1	*Ref*		*Ref*	
Yes	356	26	7.3	1.8 ( 1.2 ~ 2.8 )	0.01	2.0( 1.3 ~ 3.1 )	0.01
Syphilis infection status							
No	3 908	153	3.9	*Ref*		*Ref*	
Yes	330	33	10.0	2.7 ( 1.8 ~ 4.1 )	<0.01	2.8 ( 1.9 ~ 4.3 )	<0.01
HSV-2 infection status							
No	3 680	142	3.9	*Ref*		*Ref*	
Yes	481	36	7.5	2.0 (1.4 ~ 2.9 )	<0.01	2.3 (1.5 ~ 3.3 )	<0.01

^a^258 MSM participants were not analyzed here, in which 250 were BED-CEIA detected established HIV infected participants and 8 were HIV antibody positive participants failing to be tested by BED-CEIA for lack of sufficient samples

^b^The models were adjusted for age (continuous), study site (Shanghai, Nanjing, Changsha, Zhengzhou, Ji’nan, Shenyang and Kunming), residence (local/migrants), education (illiterate/attended primary school, junior high school, senior high school or equal/college or above) and marital status (never married/ever married)

### Factors associated with recent infection of HIV

Our study indicated that being engaged in anal sex with male partners in the last six months marginally associated with recent HIV infection (adjusted odds ratio (a*OR*) = 2.4, 95%*CI*:1.0-5.9, *P* = 0.06). Our study also pointed out that having more than two male sexual partners in last six months significantly increased the risk (a*OR* = 1.4, 95 % *CI*: 1.1-1.9) of recent HIV infection, compared to those having two or less. Compared to the respective reference groups, participants who used recreational drug (a*OR* = 2.2, 95 % *CI*: 1.6-3.0) also had significantly higher risk of recent HIV infection.

Participants who got circumcised (a*OR* = 2.0, 95 % *CI*: 1.3-3.1) or correctly answered all the eight questions about prevention of HIV (a*OR* = 1.4, 95 % *CI*: 1.0-2.0) had significantly higher chance of recent acquisition of HIV. Anal bleeding in the past six months was also found to be positively associated (a*OR* = 2.1, 95 % *CI*: 1.4-3.0) with recent HIV infection.

Risk of developing recent HIV infection was about two times higher (a*OR* = 2.8, 95 % *CI*: 1.9-4.3) among syphilis positive participants compared to their syphilis negative counterparts. HSV-2 positive participants also had significantly higher likelihood (a*OR* = 2.3, 95 % *CI*: 1.5-3.3) of developing recent HIV infection compared to those who were HSV-2 negative testing result.

## Discussion

The characteristics of HIV infection differ considerably between recently-infected and established cases. The probability of transmission of the infection also remains much higher from recently infected cases compared to the established cases [10]. However, efforts to determine the correlates of recent acquisition of HIV were limited, except one previous study that compared the predictors of HIV prevalence and incidence cases [11].Given the important role of recent HIV infection in HIV spread, in this current study we explored the role of the potential correlates of recent HIV infection among MSM in China.

Corroborating with our previous findings, we found that recreational drug use was not only positively associated with established HIV infection [12] but also positively correlated with recent acquisition of HIV among MSM. Although there were some other evidences in prior literature that recreational drug use was a positive correlate of risky sexual behavior in this population elsewhere [13], except our one previous observation this factor was rarely studied in China [12]. Thus keeping the cross-sectional design of our study in consideration, current observation highlighted the need for further exploration of the interrelationship between recreational drug use, risky sexual behaviors and risk of HIV acquisition among Chinese MSM in a prospective study.

In our study, we found that circumcision was positively associated with recent HIV infection. While male circumcision was found to reduce the risk of HIV transmission from women to men through heterosexual contacts [14], sufficient evidence was not available in favor of that male circumcision could protect against HIV infection among MSM [15]. Despite some contradictory evidences, there were also a few prior findings corroborating with our observation suggesting positive association between circumcision and recent HIV infection among MSM subgroup who predominantly practice insertive anal intercourse with male partners [16]. There might be several potential explanations for this observation. First, different from previous studies, our study focused on recent HIV infection, not established infection. Among these two types of infections, role of the correlates of HIV acquisition including that of circumcision might be different. Second, in comparison with other countries with higher rates of male circumcision rate, male circumcision well accepted MSM in China [17]. As a result, only a small proportion of MSM usually get circumcised and the majority of them undergo circumcision at a relatively older age. Potential problems associated with late circumcision include longer healing time, more frequent complications and post-circumcision fragility of skin [18]. Cumulatively these issues could well have culminated in relatively higher probability of HIV acquisition through unprotected sexual activities. MSM who were circumcised might also be different from those who were not circumcised, in terms of other predictors of HIV. This differential rate of circumcision might have resulted in variation in the observation regarding the role of circumcision in HIV transmission. Third, behavioral disinhibition could also be a potential explanation for this observation, as some circumcised MSM might have been engaged in more risky sexual behaviors after the procedure as a result of their knowledge regarding its protective role against HIV acquisition.

It was not surprising to find anal bleeding as a potential risk factor for recent HIV infection in our study. Although this factor was not well studied in China before, still there were some prior evidences corroborating with this finding [19, 20]. For participants engaged in unprotected anal sex, broken rectal or anal mucosa could have facilitated the probability of HIV acquisition in great extent [20].

Contradicting prior studies, which consistently found no association between knowledge regarding HIV prevention and risk of HIV acquisition among study participants [21], in our study those who correctly answered all the eight questions related to the prevention of HIV seemed to have higher risk of recent HIV infection. This positive correlation could be due to the difference in outcome measure (established vs. recent HIV infection) between prior studies and current one. While our findings indicated towards the existence of a huge gap between knowledge and practice regarding HIV prevention among MSM, selection bias and reverse causation (owing to temporal ambiguity) resulting respectively from convenience sampling strategy and cross-sectional design could also be somewhat responsible for the observation.

Similar to previous studies [22], we also found syphilis and HSV-2 patients were more likely to have recent HIV infection. This observation probably implied further that screening and treatment for STIs should be combined with other comprehensive intervention strategies for HIV prevention [23].

Our study also indicated towards a gradual upsurge of HIV epidemic among MSM in China amounting to a major public health concern. Prevalence proportion and incidence rate was estimated to be higher than the recently reported values obtained from the integration of surveillance data and findings from a systematic review regarding occurrence and burden of HIV among Chinese MSM [24]. Despite extensive implementation of several intervention strategies to improve the HIV scenario among MSM in China, results of this comprehensive cross-sectional study probably indicated that further expansion of the intervention strategies, including but not limiting to condom promotion, HIV testing and counseling along with increased coverage of HIV/STDs treatment were the need of the hour.

Proportion of recent HIV infection was also found to be very high. Given the higher viral load, resultant enhanced potential for HIV transmission [25], and observed high risk behaviors among these recent HIV cases, aggravating of the current HIV epidemic among Chinese MSM seemed very likely.

Our study has several limitations. As acute infection has not been referred to in China CDC formula, thus the estimated incidence is incidence of recent HIV infection, not real incidence. Also, due to the cross-sectional design of this observational study, temporal ambiguity did limit our ability to explore the causal relationship between potential predictors and recent HIV infection. For example, as we mentioned before, the positive correlation between correct knowledge and recent HIV infection could be due to temporal ambiguity and resultant reverse causation, as recently infected HIV patients among our participants, might have gathered more knowledge regarding HIV by active efforts, being influenced by the perception of their symptoms. Convenience sampling and non-participation could have introduced some amount of selection bias although this potential vulnerability was minimized by extensive efforts to increase representativeness and decrease non-participation. Social desirability bias was also a potential weakness owing to the collection of sensitive information like socio-economics, marital status, drug use, sexual behaviors etc.), through face-to-face interviews. The HIV incidence reported in our study should be interpreted with caution, as the late phase of the HIV infection stage, low CD4^+^ T cell counts, and treated with antiretrovirals (ARV) can lead the problem of misclassification.

To minimize the aforementioned sources of bias, all the reviewers were trained uniformly by long, rigorous training sessions, while the same study protocol was followed in each study site. Quality of data was also ensured by on site checking of each filled-in questionnaire by two separate faculties while the respective respondent was still present in the study site. Although we did not follow entirely RITA proposed by WHO, we adjusted for its FPR, and with aims to minimize BED-CEIA correlated FPR, we only included MSM participant who never be tested for HIV positive before. Additionally, we provided the sensitivity/specificity adjusted HIV incidence, based on China specific BED-CEIA testing window period, FPR and other authoritative parameters recommended by China CDC.

## Conclusions

Based on the findings of this comprehensive, multisite investigation, we could conclude that HIV new epidemic among Chinese MSM was very severe with an alarming number of recently infected HIV patients along with high burden of STIs. Interventions specifically targeting high-risk MSM especially those having high-risk behaviors (especially multiple partners and recreational drug use), syphilis or HSV-2 infection and anal bleeding were urgently required for efficient control of HIV among MSM in China.

## Abbreviations

aOR, adjusted odds ratio; ARV, antrovirals; BED-CEIA, BED HIV-1 capture enzyme immunoassay; ELISA, enzyme-linked immunosorbent assay; FRP, false positive rate; HSV-2, herpes simplex virus-2; MSM, men who have sex with men; RITA, recent infection testing algorithm; RPR, rapid plasma regain; STD, sexually transmitted disease; TPPA, treponema pallidum particle assay

## Additional file

Additional file 1: Multilingual abstracts in the six official working languages of the United Nations. (PDF 763 kb)
