# Supplementary material for: High HIV incidence epidemic among men who have sex with men in china: results from a multi-site cross-sectional study
Source: Infect Dis Poverty. 2016 Sep 5;5(1):82. doi: 10.1186/s40249-016-0178-x (PMC5011347; doi:10.1186/s40249-016-0178-x)
Supplement: Additional file 1: — Multilingual abstracts in the six official working languages of the United Nations. (PDF 763 kb) [file 40249_2016_178_MOESM1_ESM.pdf]

Translation of the abstract into the six official working languages of the United Nations

**وباء ارتفاع معدلات الإصابة بفيروس نقص المناعة البشرية بين الرجال الذين يمارسون الجنس مع الرجال في الصين: نتائج من دراسة متعددة المواقع مشتركة بين القطاعات**


چون چي شو، وى مينج تانج، هوا تشون زو، تانماي ماهاباترا، تشينج هاي هو، چينج فنج فو، زهي وانج، لين لو، مينج هوا تشوانج، شي تشن، چي هوا فو، يان تشيو يو چين شين لو، يونج چون چيانج، ون تشينج چنج، شياو شو هان، هونج شانج



**ملخص**

**خلفية:** يمثل ارتفاع معدلات الإصابات الجديدة بفيروس نقص المناعة البشرية بين الرجال الذين يمارسون الجنس مع الرجال (MSM) مصدر قلق كبير في الصين. وتحث قلة المعلومات على المستوى الوطني فيما يتعلق بهذا الوباء والتنبؤ بالإصابات الجديدة بالمرض على إجراء دراسة متعددة المواقع، تجرى في وقت مناسب وتكون شاملة.

**الطرق:** تم استخدام الأساليب المختلطة لفحص رجال يمارسون الجنس مع الرجال من سبع مدن في الصين بين عامي 2012 و 2013. وقدرت الإصابات الأخيرة المؤكدة من فيروس نقص المناعة البشرية عن طريق اختبار ويسترن ومقايسة المُمْتَزّ المناعي المرتبط بالإنزيم BED HIV-1. تم اختبار أيضا الزهري وفيروس الهربس البسيط 2 (HSV-2).

**النتائج:** تم فحص ما مجموعه 4496 من الرجال المؤهلين الذين يمارسون الجنس مع الرجال. كان عمر أغلبية ≤35 سنة (77.5٪)، مهاجرين (60.3٪)، لم يتزوجون أبدا (69.8٪)، لعبوا دور المتقبل في الجنس الشرجي (70.5٪). وبلغت نسبة انتشار فيروس نقص المناعة البشرية نسبة 9.9٪، منهم 41.9٪ أصيبوا مؤخرا، مع تعديل حساسية/خصوصية الإصابة بفيروس نقص المناعة البشرية 8.9 (95٪ CI: 7.6 حتى 10.2)/100 شخص-سنوات. وكانت نسبة تاريخ انتشار فيروس الهربس البسيط 2 والزهري 12.5٪ و 8.5٪ على التوالي. وارتبطت الإصابة بفيروس نقص المناعة البشرية في الآونة الأخيرة مع وجود عدة شركاء من الرجال (aOR = 1.4، 95٪ CI1،1-1.9)، تعاطي المخدرات الترفيهية (aOR = 2.2، 95٪ CI1.6-3,0)، نزيف شرجي (aOR = 2.1، 95٪ CI1,4-3.0)، إصابة بمرض الزهري (aOR = 2.8، 95٪ CI1.9-4.3) تاريخ إصابة فيروس الهربس البسيط 2 (aOR = 2.3، 95٪ CI1.5-3.3).

**الاستنتاجات:** يحتمل أن يؤدي ارتفاع معدل الإصابة بفيروس نقص المناعة البشرية الأخير إلى تدهور تدريجي في وباء فيروس نقص المناعة البشرية بين الرجال الذين يمارسون الجنس مع الرجال بشكل كلي في الصين.ويظهر أن التدخلات الهادفة لمعالجة الرجال الذين يمارسون الجنس مع الرجال المعرضين لنسبة عالية من المخاطر والذين يشملون من لديهم عدة شركاء، ومن لديهم تاريخ من تعاطي المخدرات الترفيهية والمصابين بالزهري أو عدوى فيروس الهربس البسيط 2 هي حاجة ملحة في الوقت الراهن.


Translated from English version into Arabic by Mahmoud Sami, through

TRANSLATORS
WITHOUT BORDERS

**中国男男性接触者中存在高水平的 HIV 新发感染率：来自一项多中心横断面调查的研究结果**


徐俊杰, 唐卫明, 邹华春, Tanmay Mahapatra, 胡清海, 傅更锋, 王哲，陆林，庄明华, 陈曦, 傅继华, 虞炎秋, 陆金鑫, 姜拥军, 耿文清, 韩晓旭, 尚红



**摘要**

**引言：**男男性接触者(MSM)人群 HIV 感染率逐年攀升已成为中国的一个严峻公共卫生问题。然而，针对中国 HIV 新发感染率疫情现状的进展及其影响因素尚缺乏全国水平的数据支持，亟需开展一项多中心和及时综合的调查研究予以揭示。

**方法：** 2012-2013 年，本项目在中国 7 个城市采用多种方法招募 MSM 人群开展了一项横断面调查。采用蛋白印迹法和 BED 捕获酶联免疫实验（BED-CEIA）估计 HIV 感染者的新近感染或长期感染状态。同时对入选者进行梅毒和 II 型单纯疱疹病毒（HSV-2）抗体检测。
**结果：**本研究共招募符合入选标准的 MSM 研究对象 4496 名，其中大多数 MSM 年龄≤35 岁（77.5%）、为流动人口（60.3%）、处于单身状态（69.8%），和在肛交行为中为“被插入角色”（70.5%）。该人群的 HIV 感染率为 9.9%，其中 41.9% 为 HIV 新近感染者，经校正后的 HIV 发病率为 8.9（95%*CI*: 7.6-10.2）/100 人年。HSV-2 和梅毒感染率分别为 12.5%和 8.5%。多因素 logistic 回归分析结果显示，多性伴(a*OR*=1.4, 95%*CI* 1.1-1.9)、娱乐性药物使用史(a*OR*=2.2, 95%*CI* 1.6-3.0)、肛门出血(aOR=2.1, 95%CI 1.4-3.0), 感染梅毒(a*OR*=2.8, 95%*CI* 1.9-4.3)和 HSV-2 感染史(a*OR*=2.3, 95%*CI* 1.5-3.3)是该人群 HIV 新近感染的独立影响因素。
**结论：**MSM 人群较高的 HIV 新近感染率是导致中国 HIV 总体疫情持续恶化的潜在影响因素。因此当务之急需对具有高危特征突出的 MSM 人群，如具有多性伴、娱乐性药物使用史、梅毒或 HSV-2 感染的亚人群开展针对性的干预措施。

Translated from English version into Chinese by Jun-jie Xu, Yan-qiu Yu

**Forte augmentation de l’incidence du VIH chez les hommes homosexuels : résultats d’une étude transversale multisites**

Jun-Jie Xu, Wei-Ming Tang, Hua-Chun Zou, Tanmay Mahapatra, Qing-Hai Hu, Geng-Feng Fu, Zhe Wang, Lin Lu, Ming-Hua Zhuang, Xi Chen, Ji-Hua Fu, Yan-Qiu Yu, Jin-Xin Lu, Yong-Jun Jiang, Wen-Qing Geng, Xiao-Xu Han, Hong Shang

**Résumé**

**Contexte:** La récente recrudescence des nouveaux cas d’infections au VIH chez les hommes homosexuels est une cause d’inquiétude majeure en Chine. Le manque d’informations au nationales concernant le fardeau et les indicateurs de cette épidémie croissante de nouveaux cas nécessitait une enquête multicentrique et complète et menée dans un bref délai.
**Méthodes:** Des méthodes combinées ont été utilisées pour recruter des hommes homosexuels dans sept villes de Chine entre 2012 et 2013. Les infections par le VIH récentes et établies ont été estimées par Western Blot et dosage immunoenzymatique BED HIV-1. La syphilis et les infections par le virus de l’herpès VHS-2 ont aussi été recherchées.
**Résultats:** 4496 hommes homosexuels éligibles ont été recrutés. La majorité avaient 35 ans ou moins (77,5 %), étaient migrants (60,3 %), n’avaient jamais été mariés (69,8 %), et avaient un rôle passif lors des rapports sexuels anaux (70,5 %). La prévalence du VIH était de 9,9 % et 41,9 % de ces cas avaient été récemment infectés, avec une incidence du VIH ajustée par la sensibilité/spécificité de 8,9/100 personnes par an (IC à 95 % : 7,6-10,2). La prévalence des infections anciennes par le VHS-2 et la syphilis était respectivement de 12,5 % et 8,5 %. Les infections par le VIH récentes étaient associés au fait d’avoir plusieurs partenaires masculins (ORa=1,4, IC à 95 % 1,1-1,9), à la consommation de drogues (ORa=2,2, IC à95 % 1,6-3,0), aux saignements anaux (ORa=2,1, IC à 95 % 1,4-3,0), aux infections par la syphilis (ORa=2,8, IC à 95 % 1,9-4,3) et aux infections passées par le VHS-2 (ORa=2,3, IC à 95 % 1,5-3,3).
**Conclusions:** Le taux élevé d’infections récentes par le VIH pourrait causer une aggravation de l’épidémie générale de VIH parmi les homosexuels en Chine. L’important à l’heure actuelle est d’entreprendre des

interventions ciblées afin d'informer les hommes homosexuels à haut risque, notamment ceux qui ont plusieurs partenaires sexuels, des antécédents de toxicomanie, et qui ont été infectés par la syphilis ou le VHS-2.

Translated from English version into French by Suzanne Assenat, through

**Высокая частота заражения ВИЧ носит эпидемических характер среди мужчин, имеющих половые связи с мужчинами в Китае: Результаты исследования различных регионов и различных групп населения**

Юн-Джи Ху, Вей-Минг Танг, Хуа-Чун Жоу, Танмей Махапатра, Цин-Хай Ху, Женг-Фен Фу, Же Ванг, Лин Лу, Минг-Хуа Жуанг, Ксиі Чен, Джи-Хуа Фу, Ян-Ци Юу, Йин-Син Лу, Йонг-Йун Джанг, Вен-Цин Дженг, Ксао-Ксу Хан, Хонг Шанг

**Краткое описание ситуации**

**Общие сведения:** Учащение новых случаев заражения ВИЧ в последнее время среди мужчин, имеющих половые связи с мужчинами, является основной проблемой в Китае. Недостаток информации на национальном уровне относительно тяжести последствий и предпосылок прогрессирующей эпидемии новых случаев заражения потребовало проведения своевременного и тщательного исследования в различных очагах инфекции.

**Методы:** Смешанные методы использовались, чтобы привлечь мужчин, имеющих половые связи с мужчинами в семи городах Китая в 2012-2013 годах. Новые и давние случаи заражения ВИЧ были исследованы по методу Вестерн-блот и имуннoферментного анализа. Также были проведены тесты на сифилис и вирус герпеса 2 типа.

**Результаты:** Было привлечено всего 4496 мужчин, имеющих половые связи с мужчинами, отвечающих критериям отбора. Большинство из них было в возрасте младше 35 лет (77,5%), мигранты (60,3%), никогда не были женаты (69,8%), и играли пассивную роль при анальном совокуплении (70,5%). Частота заражения ВИЧ составила 9,9%, и 41,9% были недавно инфицированы, с скорректированной частотой инфицирования ВИЧ по чувствительности/специфичности в 8,9 (95% 7,6-10,2)/100 человеколет. Частота предшествующего заражения вирусом герпеса 2 типа и сифилисом составило 12,5% и 8,5% соответственно. Недавнее инфицирование ВИЧ было связано с наличием половых контактов с несколькими мужчинами (a*OR*=1.4, 95%*CI* 1.1-1.9), употреблением наркотиков в целях времяпровождения (a*OR*=2.2, 95%*CI* 1.6-3.0), анальным кровотечением (a*OR*=2.1, 95%*CI* 1.4-3.0), инфекцией сифилиса (a*OR*=2.8, 95%*CI* 1.9-4.3) и предшествующим заражением вирусом герпеса 2 типа (a*OR*=2.3, 95%*CI* 1.5-3.3).

**Выводы:** Высокий уровень новых случаев инфицирования ВИЧ может быть результатом ухудшающейся эпидемической ситуации среди мужчин, имеющих половые контакты с мужчинами в Китае. Целевое воздействие, адресованное к мужчинам, имеющим половые контакты с мужчинами, составляющим группу риска, включая тех, кто имеет несколько партнёров, в прошлом употребляли наркотики в целях времяпровождения или были инфицированы сифилисом или вирусом герпеса типа 2, представляется острой необходимостью.

Translated from English version into Russian by Tatiana Glazina, through

**Alta incidencia de epidemia de VIH entre hombres que tienen sexo con otros hombres en China: Resultados de un estudio transversal multisitio**

Jun-Jie Xu, Wei-Ming Tang, Hua-Chun Zou, Tanmay Mahapatra, Qing-Hai Hu, Geng-Feng Fu, Zhe Wang, Lin Lu, Ming-Hua Zhuang, Xi Chen, Ji-Hua Fu, Yan-Qiu Yu, Jin-Xin Lu, Yong-Jun Jiang, Wen-Qing Geng, Xiao-Xu Han, Hong Shang

**Resumen**

**Antecedentes:** El reciente surgimiento de nuevas infecciones por VIH entre hombres que tienen sexo con otros hombres es de gran preocupación en China. La escasez de información a nivel nacional en cuanto a la cargas y los indicadores de esta epidemia progresiva de nuevas infecciones requirió de una investigación multicéntrica, oportuna e integral.

**Métodos:** Se utilizaron métodos combinados para reclutar a hombres que tenían sexo con otros hombres en China entre los años 2012 y 2013. Se estimaron las infecciones por VIH recientes y las ya establecidas mediante Western Blot e inmunoensayo de captura de enzimas BED VIH-1. También se evaluó la presencia de sífilis y virus del herpes simple-2 (HSV-2).

**Resultados:** Se reclutó un total de 4496 hombres elegibles que tenían sexo con otros hombres. La mayoría tenían ≤35 años de edad (77,5%), eran inmigrantes (60,3%), solteros (69,8%) y jugaban un rol receptivo en el sexo anal (70,5%). La prevalencia de VIH fue del 9,9% y 41,9% habían sido recientemente infectados, con incidencia de sensibilidad/especificidad ajustada de VIH del 8,9 (95%*IC*: 7,6-10,2)/100 Personas-Años. La prevalencia de antecedentes de HSV-2 y sífilis fue de 12,5% y 8,5%, respectivamente. La infección reciente por VIH estaba asociada al tener múltiples parejas masculinas (a*OR*=1,4, 95%*IC* 1,1-1,9), el uso de drogas recreativas (a*OR*=2,2, 95%*IC* 1,6-3,0), sangrado anal (a*OR*=2,1, 95%IC 1,4-3,0), infección por sífilis (a*OR*=2,8, 95%*IC* 1,9-4,3) y antecedentes de infección por HSV-2 (a*OR*=2,3, 95%*IC* 1,5-3,3).

**Conclusiones:** El elevado índice de infección reciente por VIH está potencialmente resultando en un deterioro progresivo de la epidemia general de VIH entre hombres que tienen sexo con otros hombres en China. La necesidad del momento parecen ser intervenciones dirigidas hacia hombres de alto riesgo que tienen sexo con otros hombres, incluyendo aquellos que tienen múltiples parejas, antecedentes de uso de drogas recreativas y sífilis o infección por HSV-2.

Translated from English version into Spanish by Maria Alejandra Aguada, through
